# Metabolomic Patterns at Birth of Preterm Newborns with Extrauterine Growth Restriction: Towards Putative Markers of Nutritional Status

**DOI:** 10.3390/metabo15080518

**Published:** 2025-08-01

**Authors:** Marta Meneghelli, Giovanna Verlato, Matteo Stocchero, Anna Righetto, Elena Priante, Lorenzo Zanetto, Paola Pirillo, Giuseppe Giordano, Eugenio Baraldi

**Affiliations:** 1 Neonatal Intensive Care Unit, Department of Women’s and Children’s Health, University Hospital of Padova, 35128 Padova, Italy; anna.righetto@studenti.unipd.it (A.R.); elena.priante@aopd.veneto.it (E.P.); lorenzo.zanetto@studenti.unipd.it (L.Z.); eugenio.baraldi@unipd.it (E.B.); 2 Paediatric Nutrition Service-Neonatal Intensive Care Unit, Department of Women’s and Children’s Health, University Hospital of Padova, 35128 Padova, Italy; 3 Institute of Pediatric Research “Città della Speranza”, Department of Women’s and Children’s Health, University of Padova, 35127 Padova, Italy; matteo.stocchero@unipd.it (M.S.); paola.pirillo@unipd.it (P.P.); giuseppe.giordano@unipd.it (G.G.)

**Keywords:** metabolomics, extrauterine growth restriction, very low birth weight preterm newborns

## Abstract

**Background:** Nutrition is of paramount importance during early development, since suboptimal growth in this period of life is linked to adverse long- and mid-term outcomes. This is particularly relevant for preterm infants, who fail to thrive during the first weeks of life and develop extrauterine growth restriction (EUGR). This group of premature babies represents an interesting population to investigate using a metabolomic approach to optimize nutritional intake. **Aims:** To analyse and compare the urinary metabolomic pattern at birth of preterm infants with and without growth restriction at 36 weeks of postmenstrual age or at discharge, searching for putative markers of growth failure. **Methods:** We enrolled preterm infants between 23 and 32 weeks of gestational age (GA) and/or with a birth weight <1500 g, admitted to the Neonatal Intensive Care Unit (NICU) at the Department of Women’s and Children’s Health of Padova University Hospital. We collected urinary samples within 48 h of life and performed untargeted metabolomic analysis using mass spectrometry. **Results**: Sixteen EUGR infants were matched with sixteen non-EUGR controls. The EUGR group showed lower levels of L-cystathionine, kynurenic acid, L-carnosine, N-acetylglutamine, xanthurenic acid, aspartylglucosamine, DL5-hydroxylysine-hydrocloride, homocitrulline, and L-aminoadipic acid, suggesting a lower anti-inflammatory and antioxidant status with respect to the non-EUGR group. **Conclusions:** Metabolomic analysis suggests a basal predisposition to growth restriction, the identification of which could be useful for tailoring nutritional approaches.

## 1. Introduction

The impact of nutrition and growth during the very early stages of life is a topic of great interest, especially for preterm newborns, born with a birth weight of less than 1500 g (Very Low Birth Weight Infants, VLBWI). The early parenteral (PN) and enteral nutritional support received by VLBWI during the Neonatal Intensive Care Unit (NICU) usually does not match fetal nutrient intakes. This can result in protein and energy deficits lasting for weeks, and potentially promoting the subsequent postnatal growth restriction, to which also non-nutritional factors contribute [1,2]. In order to face growth failure, research has focused on best feeding strategies [3] and has developed guidelines to standardize and enhance the nutritional care of VLBWI both during and after their stay in the NICU [4,5].

Ensuring adequate growth after birth for VLBWI is mandatory for their present and future health, as it seems that malnutrition during early life and consequently the related “thrifty phenotype” could lead to cardiometabolic syndrome later in life [6]. However, we still do not know which is the best nutritional approach. We also lack reliable biomarkers that can reflect or predict the risk of growth failure during hospitalization.

Extrauterine growth restriction (EUGR) is a condition of inadequate growth during hospitalization, and it is a common outcome among VLBW preterm infants. There are several definitions of EUGR in use today, mainly cross-sectional definition (weight at a given time <10th percentile), or a longitudinal one (weight loss between birth and a given time with different standard deviation (SD) thresholds, usually > 1 SD) [7,8,9]. Growth assessment is performed using anthropometric charts based on cross-sectional or longitudinal data [10,11].

EUGR may result from many factors: the development of significant protein and energy deficit during the first weeks of life—which is difficult to reverse [1]—male gender, intrauterine growth-restricted (IUGR) or small for gestational age (SGA) condition, prolonged need for respiratory support, and the development of neonatal morbidities [12,13].

The incidence is variable depending on the definition used, the population considered, and the characteristics of the centre, but it is a significant issue worldwide. For example, data from the Neonatal Research Network of the National Institute of Child and Human Development (NICHD), collected from extremely low GA (22–28 weeks) and VLBWI (401–1500 g), reported growth failure (weight < the 10th percentile for gender at PMA of 36 weeks), in 79% of infants [14]. Other studies reported a lower incidence of 28–51% [9,15]. Interestingly, a recent study (with a population of 2514 very preterm infants) developed a real-time risk prediction model using eight factors: birth weight, SGA, hypertensive disease complicating pregnancy, gestational diabetes mellitus, multiple births, cumulative duration of fasting, growth velocity, and postnatal corticosteroids to predict the probability of risk of EUGR in preterm infants born at gestational age < 32 weeks of GA [16].

Taking into account the importance of adequate early growth [17], the implementation of standardized feeding guidelines allowed to show a reduction in the incidence of EUGR. Nonetheless, intensive nutritional support does not work for everyone, and even when extremely premature infants reach a growth velocity rate that is within current guidelines, growth restriction remains high [18]. Therefore, investigating the mechanisms leading to postnatal suboptimal growth is challenging and could contribute to improving clinical practices.

Metabolomics may help in discovering putative markers of nutritional and clinical status in preterm newborns, which may improve and individualize nutritional plans. Indeed, exploratory studies using untargeted metabolomic approaches allow for both the qualitative and quantitative determination of thousands of different metabolites within a biological sample. This approach could help identification of putative markers and metabolic patterns characteristic of a given condition [19] and could be applied to identify patients at risk of suboptimal growth.

Recently, the metabolic pathways implicated in EUGR pathophysiology were investigated. Dudzik et al. found significantly reduced levels of amino acids and phospholipids in the plasma of EUGR patients born at <32 weeks of GA, while Duan et al., in preterm infants less than 34 weeks of GA (but excluding SGA or VLBW), identified a total of 58 and 71 differential metabolites in fecal and plasma samples, respectively, involved in pathways such as caffeine, galactose, glutathione, cysteine, and methionine metabolisms [20,21].

In our previous study [22], we showed that neonates with intrauterine growth restriction (IUGR) showed a distinctive urinary metabolic profile at birth.

Untargeted metabolomic suggested clear clustering of IUGR neonates compared to controls, indicating a possible disruption in pathways linked to metabolism of histidine and tryptophan, as well as aminoacyl-tRNA and steroid hormone biosynthesis [22]. Applying a similar approach, we present here a new exploratory pilot study aimed at analysing and comparing the urinary metabolomic pattern at birth of preterm infants with and without growth restriction at 36 weeks of postmenstrual age or at discharge, searching for putative markers of growth failure during early life.

## 2. Materials and Methods

### 2.1. Study Population

In this prospective, monocentric observational pilot study, we enrolled preterm infants between 23 and 32 weeks of GA and/or with a birth weight <1500 g born and then admitted to the Neonatal Intensive Care Unit (NICU) of the Department of Women’s and Children’s Health at Padova University Hospital from October 2021 to October 2023.

Exclusion criteria comprised infants with major congenital abnormalities or chromosomal abnormalities, with known or suspected congenital metabolic diseases, those with hemodynamic instability not allowing early enteral feeding, those experiencing asphyxia, newborns who had received transfusions before urine collection, and those with refusal of consent. For each eligible subject, the following clinical data were collected: gestational age, birth weight, sex, multiple pregnancy, mode of delivery, antenatal steroids administration, Apgar score at 5 min, IUGR, bronchopulmonary dysplasia (BPD) diagnosis, early onset sepsis (EOS), late onset sepsis (LOS), retinopathy of prematurity (ROP) grade, days to full enteral feeding, weight percentile at birth, and at 36 weeks of postmenstrual age (GA) or at discharge (if ≤10th percentile the patient was defined as SGA at birth and EUGR at 36 weeks of GA or discharge).

All included patients received the same nutritional regimen according to the internal protocol. A progressive total parenteral nutrition infusion was initiated at admission through a central umbilical or percutaneous line, starting with 6–8 g/kg/day of glucose and 1.5 g/kg/day of amino acids with the target to achieve in the following days a non-protein energy >80 kcal/kg/day (from glucose and lipids) and at least 3.5 g/kg/day of amino acids. Starting from birth to the second day of life, patients received minimal enteral feeding of 10–20 mL/kg/day with mother’s own milk or donor human milk, then increased by 10–20 mL/kg/day according to our nutritional protocol.

Ethical approval for the study was obtained from the Ethics Committee of Padua Hospital (protocol number: 4374/AO/17). All the enrolled patients had a written informed consent for participation from a legally acceptable representative.

### 2.2. Sample Collection and Preparation

At least 2 mL of urine was collected from each enrolled patient within 48 h of life. Urine samples were collected using a non-invasive method involving a sterile cotton swab placed inside the infant’s diaper, with checks for urine presence every 30 min. A sterile glove was interposed between the cotton and the diaper to reduce the risk of contamination. The cotton swab was changed every 3 h if the infant did not urinate or if it became contaminated with faecal material. Once the swab was saturated, the cotton was placed inside a sterile 20 cc syringe, compressed mechanically using the plunger, and the extracted urine was transferred into a sterile container pre-washed with methanol (MeOH). The samples were stored at −80 °C until metabolomic analysis.

### 2.3. Metabolomic Analysis

Untargeted metabolomics analysis was performed at the Mass Spectrometry and Metabolomics Laboratory of the Istituto di Ricerca Pediatrica, in the Women’s and Children’s Health Department of the Padova University.

The same experimental procedures reported in Priante et al. [22] were applied. Specifically, urine samples were slowly thawed overnight at +4 °C and then brought to room temperature. Each sample was mixed and centrifuged at 3600× *g* for 10 min at 10 °C. Subsequently, 60 µL of the supernatant from each sample was transferred to a 384-well plate, with the addition of 240 µL of 0.1% formic acid (FA) solution (final volume 300 µL, 1:5 dilution). All preparation procedures were automated using a robotic sample handling system, the Multiprobe II Ex (Perkin Elmer, Waltham, MA, USA).

Then, an untargeted metabolomic profile of the urine samples was obtained in both positive and negative ionization modes using an Acquity Ultra Performance Liquid Chromatography (UPLC) system (Waters, Manchester, UK) coupled with a high-resolution mass spectrometer, specifically the Synapt G2 HDMS (Waters MS Technologies, Ltd., Manchester, UK). More details about the chromatographic method and mass detection can be found in Priante et al. [22].

Quality control (QC) samples and standard solution samples (Mix) were utilized to assess reproducibility and accuracy during the analysis and to examine metabolite content of the samples. The Mix consisted of nine compounds with known exact masses and retention times, injected under the same conditions as the samples to determine retention time and mass in the specified analytical conditions. QC and Mix were injected at regular intervals, every 15 samples, during the sequence, alongside blank samples (0.1% FA aqueous solution) to identify specific ions from the mobile phase and any contaminants.

Data were pre-processed using Progenesis QI software (Waters Corporation, Milford, USA), generating two datasets: one for positive ionization mode (ESI+ dataset) and another for negative ionization mode (ESI− dataset). Parameters for data extraction were optimized through preliminary processing of QC samples. A filter of 0.5 and 0.2 was set to import raw data from the samples into Progenesis for positive and negative ionization modes, respectively, with a QC sample positioned centrally in the sequence as a reference for automatic alignment of all runs. The sensitivity of the automatic algorithm for peak-picking was set to 5, within a chromatographic range of 0.4 to 8.0 min.

Variables with missing data in the QCs or with more than 10% of missing data in the samples were excluded. Missing data were imputed by extracting a random number from a uniform distribution between zero and the minimum value recorded for the variable. Moreover, variables with a coefficient of variation in the QCs exceeding 20% were excluded. Probabilistic quotient normalization was applied to mitigate the effects of sample dilution. Variables were annotated after searching our in-house database, resulting in a level of annotation equal to 1 [23]. Annotated variables were autoscaled prior to performing data analysis.

### 2.4. Statistical Data Analysis

Clinical characteristics of the enrolled patients were investigated using the *t*-test or Mann–Whitney test in the case of normally or non-normally distributed data, respectively, while qualitative data were investigated via Fisher’s exact test. A significance level of 0.05 was assumed. Normality was assessed using the Shapiro–Wilk, test, assuming normally distributed data for the *p*-value (p) greater than 0.10.

To avoid bias due to confounding factors in the data analysis, a matching procedure was implemented to select two groups of neonates, EUGR (case) and non-EUGR (control), from the eligible neonates [22]. Specifically, a distance matrix between the eligible neonates of EUGR and of non-EUGR groups was calculated considering all the clinical data recorded, excluding weight and z-score weight at 36 weeks GA. For each EUGR neonate, the non-EUGR neonate with the minimum distance was selected, and the pairs of case-control obtained were sorted on the basis of increasing distance. Pairs with the greatest distance were iteratively excluded until the set of selected EUGR neonates and that of selected controls showed the *p*-values greater than 0.05 in their clinical data.

Both multivariate and univariate data analyses were conducted. Specifically, Principal Component Analysis (PCA) was used for exploratory data analysis and outlier detection, whereas PLS for classification (PLS2C) [24] was employed for comparing the two groups under investigation. Stability selection based on variable influence on projection was performed to discover relevant and irrelevant variables [25]. In stability selection, 200 subsets were extracted using Binary Matrix Sampling with a probability equal to 0.70 for both observations and variables, and a significance level of 0.05 was assumed for the selection of relevant and irrelevant features. The number of score components was assessed on the basis of the first maximum of the Matthews correlation coefficient (MCC), calculated using 10-repeated 5-fold cross-validations under the condition to pass permutation test on the class (1000 random permutations).

For univariate data analysis, logistic regression and the Mann–Whitney test were applied. To avoid losing interesting metabolites, we considered metabolites with both *p*-value of the regression coefficient of the logistic regression model and *p*-value of the Mann–Whitney test less than 0.10 as relevant.

Data analysis was conducted using in-house R functions implemented on the R 4.2.2 platform (R Foundation for Statistical Computing).

## 3. Results

Out of a cohort of 139 eligible newborns at birth, 76 met the exclusion criteria (congenital disease, haemodynamic instability, confirmed early-onset sepsis, need for blood component before urine collection, or because of missing parental consent). The other 14 had improper urine collection. A total of 49 patients were enrolled. Among these subjects, 14 patients showed IUGR at birth, while 35 were appropriate for gestational age with normal growth in utero. Within the group of IUGR newborns, 5 were also SGA at birth, and all these patients became EUGR at 36 weeks of gestational age, together with 12 AGA newborns at birth. Therefore, at the age of 36 weeks GA (or earlier if the baby was transferred to other facilities or discharged), 26 patients had extrauterine growth restriction. The matching procedure allowed us to select 16 patients with EUGR and a matched control group of 16 non-EUGR patients. The clinical characteristics of the two groups are reported in Table 1. As expected, EUGR patients needed more days to reach full enteral feeding, while other clinical outcomes (BPD, EOS, LOS, and ROP grade) did not differ between the two groups.

**Figure 1:**
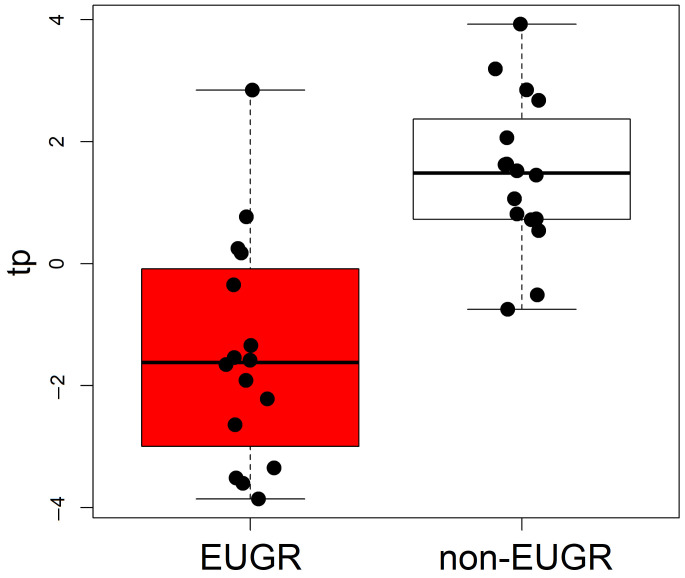
Boxplots illustrating the distribution of the predictive score tp calculated using PLS2C within the two groups compared. Black points represent the values of the score; red is used for the EUGR group whereas white for the non-EUGR group.

After data pre-processing and merging the annotated variables arising from negative and positive ionization modes, a dataset composed of 163 metabolites and 32 observations was obtained. No outliers within each group were detected through PCA on the basis of the T2 and Q-tests, assuming a significance level of 0.05. Excluding 130 irrelevant variables discovered via stability selection, the PLS2C model showed one predictive score component, MCC equal to 0.700 (*p* = 0.034), and MCC calculated using cross-validation equal to 0.645 (*p* = 0.002). The distribution of the predictive scores within the two groups compared is reported as boxplots in Figure 1. Moreover, the predictive score component was not associated with the clinical characteristics.

**Figure 2:**
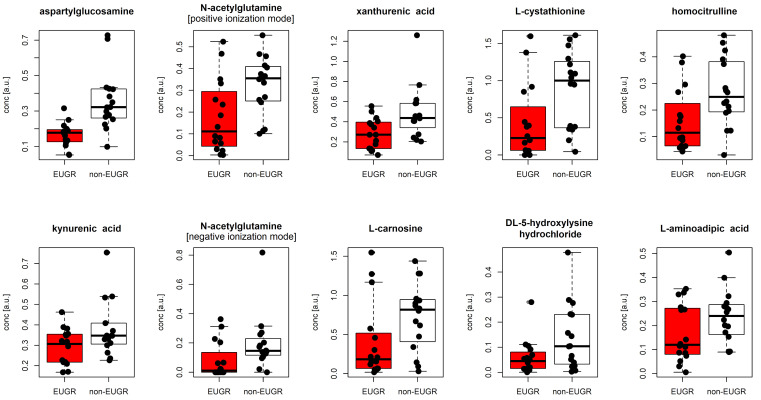
Boxplots illustrating the distributions of metabolite concentrations of the relevant metabolites. Black points represent the experimental data; red is used for the EUGR group, whereas white for the non-EUGR group.

Applying logistic regression analysis and the Mann–Whitney test to the 33 metabolites used in the PLS modelling, 10 metabolites were selected as relevant in distinguishing the two groups. The results are reported in Table 2. The distributions of the selected metabolites are reported as boxplots in Figure 2.

## 4. Discussion

EUGR remains a common complication among preterm infants despite improved clinical and nutritional care during NICU stay. As the first thousand days of life are a critical period of time, impacting long-term health, it seems crucial to better understand the mechanisms underlying growth restriction [9,14,15].

When we compared urinary metabolomic patterns at birth, the EUGR group showed lower levels of L-cystathionine, kynurenic acid, L-carnosine, N-acetylglutamine, xanthurenic acid, aspartylglucosamine, DL5-hydroxylysine-hydrocloride, homocitrulline, and L-aminoadipic acid with respect to the non-EUGR group. Specifically, some of these dysregulated pathways could be related to a lower anti-inflammatory and antioxidant status in the EUGR group. Sources of bias were minimized through a matched case-control analysis accounting for factors potentially influencing the urinary metabolome at birth.

Hereafter, we discuss possible mechanisms connecting these metabolites to growth failure.

Cystathionine is a precursor of cysteine, a semi-essential amino acid (obtained from the diet or produced through the degradation of methionine).

Cysteine plays a critical role in maintaining redox balance within cells, and it is a precursor of biologically active molecules such as glutathione, a strong antioxidant [26,27]. It is involved in a number of physiological functions, such as regulation of cell development, cell signalling, and programmed cell death (apoptosis), lipid metabolism and protein synthesis [28], and in controlling inflammation. Numerous inflammatory and oxidative stress-related disorders have been linked to decreased cysteine levels [29]. The lower cystathionine level in the urine of infants who develop growth retardation might suggest a basal unfavourable balance toward an inflammatory state. From this perspective, growth failure could be assimilated to other different pro-inflammatory morbidities of prematurity, such as RDS, chronic lung disease, and perinatal brain injury, related also to inadequate antioxidant systems [30].

Tryptophan metabolism can be divided into two main pathways: the prevalent one namely the “kynurenine shunt” (>90% of the whole tryptophan biotransformation), in which the indole ring is broken to produce compounds as kynurenines, nicotinic acid, and the nicotinamide adenine dinucleotide (NAD+), and the second one (approximately 10% of the whole tryptophan transformations), in which the indole ring is retained and tryptophan is transformed in neurotransmitters/hormones as serotonin, N-acetyl-serotonin, melatonin, and trace amines as tryptamine and derivatives. The kynurenine pathway was found to be one of the most impaired in IUGR [22,31].

A lower level of kynurenic acid might suggest a similar behaviour between IUGR and infants who develop EUGR, as the kynurenine shunt seems to be less active also in this group, suggesting a pathogenetic role in growth restriction of this pathway.

Carnosine, mainly present in skeletal muscle, is produced from histidine and beta-alanine through the hydrolysis of ATP. Studies on carnosine have demonstrated its antioxidant action, as a chelator of metal ions and as a scavenger of reactive oxygen species and peroxy-radicals [32]. It is known that the role of oxidative stress in the pathogenesis of intrauterine growth retardation, and in fact, this metabolite was detected in lower levels in the urine of IUGR infants [22,33], possibly for its consumption due to its activity as an oxidative product scavenger. The same mechanism could also be active in newborns who are destined for extrauterine growth restriction. Administering carnosine may offer antioxidative protection by lowering the concentration of oxidative damage indicators [34,35]. Recognizing carnosine deficiency at birth could identify the subgroup of preterm infants who can benefit from its supplementation.

N-acetylglutamine is the biologically available form of L-glutamine, the most abundant non-essential amino acid in our organism, that plays crucial roles in cellular metabolism and human physiology, being an energy source for cells. During stressful situations like illness, trauma, or intense exercise, glutamine is quickly used as an energy substrate. Its availability is crucial for promoting protein synthesis and cellular metabolism under these situations [36]. Glutamine also acts as an important precursor for the synthesis of nucleotides and other amino acids [37]. Another important feature of glutamine is its capacity to regulate immunological responses: it supports lymphocyte proliferation and cytokine production [38]. Thus, our finding of lower levels of N-acetylglutamine in the urine metabolome of neonates who develop growth retardation might indicate its higher use, potentially attributable to a basal state of increased catabolism.

Xanthurenic acid is a product of the tryptophan–kynurenine pathway. Disturbances in this pathway have been evaluated in a variety of clinical conditions (including immune suppression, cancer progression, and immune/inflammatory reactions, such as in IBD) [39]. The entire pathway is heavily dependent on vitamin B6 availability; therefore, xanthurenic acid has been studied as a biomarker for B6 status [40]. Functional B6 deficiency can be a consequence of dysglycemia, caused by inflammatory upregulation of the kynurenine pathway, ultimately leading to elevated levels of xanthurenic acid [41].

Homocitrulline belongs to the class of organic compounds known as l-alpha-amino acids, and it is a secondary metabolite (metabolites that are physiologically or metabolically non-essential but could function as defense or signalling molecules). A significant number of articles have been published on homocitrulline [42], which is formed nonenzymatically from lysine residues in the polypeptide chain through the action of cyanate. The latter compound is derived either from urea or from thiocyanate, via a reaction catalyzed by the enzyme myeloperoxidase (MPO). The formation of homocitrulline is known as carbamylation. In humans, this process takes place predominantly under two types of situations: uremia and inflammation [42].

Aminoadipic acid is a metabolite in the principal biochemical pathway of lysine, as an intermediate in the breakdown or degradation of lysine and saccharopine. It antagonizes neuroexcitatory activity modulated using the glutamate receptor N-methyl-D-aspartate (NMDA). Aminoadipic acid has also been shown to inhibit the production of kynurenic acid, a broad-spectrum excitatory amino acid receptor antagonist, in brain tissue slices [43]. Therefore, aminoadipic acid can act as an acidogen, a diabetogen, an atherogen, and a metabotoxin depending on the circumstances. As a diabetogen, serum aminoadipic levels appear to regulate glucose homeostasis and have been highly predictive of individuals who later develop diabetes [44].

We still need to address the meaning of a lower level of homocitrulline and aminoadipic acid, as it is for aspartylglucosamine, a member of the class compounds known as glycosylamines, and of DL5-hydroxylysine-hydrocloride, a hydroxylated derivative of the amino acid lysine that is present in certain collagens, the chief structural protein of mammalian skin and connective tissue. Further studies are needed to clarify the possible role of these metabolic pathways in preterm infants.

Our study has some significant limitations. First of all, the small sample size, mainly due to technical issues. Several urinary samples could not be used for analysis because they were not quantitatively or qualitatively adequate (i.e., inadequate amount of urine collected, sample breakage, or unsuccessful analysis). Nonetheless, our study is an exploratory pilot study since preliminary data were not available and we could not perform a prior power analysis. In any case, large-scale metabolomics studies on EUGR are still needed to validate potential biomarkers. Moreover, patient stratification based on variables such as the cause of preterm birth was not feasible due to the limited sample size. Another limitation was the absence of a concurrent plasma metabolic profile, as only urine samples were collected. Targeted blood metabolomics could be considered in future investigations. Regarding urine collection, the use of sterile cotton swabs is not ideal due to potential contamination from environmental exposure and handling. Nevertheless, this method was chosen for its extensive use [45,46], simplicity, rapid application, and to avoid skin irritation from adhesive urine bags in preterm infants. To minimize contamination, sterile devices were used throughout the procedure. Despite these precautions, refinement of the collection method should be considered in future studies. Furthermore, the 48-h window for sample collection, while necessary to account for delayed diuresis in preterm neonates, may have introduced variability related to early postnatal physiological changes and nutritional interventions. Finally, noting that some of the above-mentioned amino acids (tryptophan, methionine, cysteine, and histidine) are all exogenously provided to preterm babies through parenteral nutrition from the first hours of life, potentially influencing the urinary metabolome. Although all newborns received the same standardized nutritional regimen according to our NICU protocol, individual differences in nutrient absorption, tolerance, and metabolism may still have affected metabolite levels, representing an additional potential confounder.

## 5. Conclusions

EUGR is a common complication in VLBW preterm infants, despite numerous efforts to standardize and enhance nutritional support, which could contribute to the onset of chronic adult diseases.

A different urinary metabolomic profile at birth was discovered in EUGR infants, in part characterized by lower levels of anti-inflammation and antioxidant metabolites with respect to non-EUGR newborns. These results could suggest a sort of basal fingerprint of EUGR and may help to identify the group of newborns at risk of developing this condition, in which a tailored nutritional support, and an aggressive one, may be necessary.

Further studies are therefore needed to better understand the metabolomic profile of growth restriction to tailor prophylactic and therapeutic strategies.

## Figures and Tables

**Table 1:** Comparison of clinical characteristics of the group of 16 EUGR infants and of the 16 non-EUGR infants selected for the metabolomic investigation. Numerical data with normal distributions are reported as mean ± standard deviation, while non-normally distributed or ordinal data are presented as median (25th–75th percentile). Categorical data are expressed as counts (percentages).

Characteristic	EUGR (n = 16)	Non-EUGR (n = 16)	*p*
Birth weight, g	1026.88 ± 327.01	1111.18 ± 306.86	0.34
Gestational age, weeks	29.1 ± 2.1	28.6 ± 1.6	0.121
Male sex	6 (37.5%)	4 (25%)	0.613
Female sex	10 (62.5%)	12 (75%)	0.613
Multiple pregnancy	4 (25%)	6 (37.5%)	0.613
Cesarean delivery	14 (87.5%)	14 (87.5%)	0.678
Complete prenatal steroids	4 (25%)	8 (50%)	0.421
Apgar score at 5 min	8.0 (7.0–8.0)	8.0 (7.0–8.0)	0.658
IUGR	3 (19%)	0 (0%)	0.226
BPD	7 (43.8%)	3 (18.8%)	0.435
EOS	1 (6.3%)	1 (6.3%)	1.000
LOS	6 (37.5%)	2 (12.5%)	0.251
ROP grade	1 (0–1)	0 (0–1.5)	0.559
Days to full enteral feeding	26.9 ± 14.3	17.5 ± 8.2	<0.001
Weight at 36 weeks GA, g	1782 ± 196	2325 ± 292	<0.001
z-score weight at 36 weeks GA	−2.09 ± 0.55	−0.88 ± 0.51	<0.001

Abbreviations: EUGR = extrauterine growth restriction; GA = gestational age; IUGR = intrauterine growth restriction; BPD = bronchopulmonary dysplasia; EOS = early-onset sepsis; LOS = late-onset sepsis; and ROP = retinopathy of prematurity.

**Table 2:** Results of the data analysis: plogistic is the *p*-value of the regression coefficient in the logistic regression model; MCC and MCCcv are the Matthew correlation coefficients in calculation and in 10-repeated 5-fold cross-validation, respectively, for the logistic model; and pMW is the *p*-value of the Mann–Whitney test. Levels of the metabolites in the two groups are reported as median [IQR].

Name	plogREG	pMW	MCC	MCCcv	EUGR	Non-EUGR
aspartylglucosamine	0.005	1.87 × 10^−5^	0.689	0.683	0.177 [0.063]	0.322 [0.160]
N-acetylglutamine [positive ionization mode]	0.018	6.61 × 10^−3^	0.445	0.438	0.111 [0.226]	0.355 [0.153]
N-acetylglutamine [negative ionization mode]	0.061	1.21 × 10^−2^	0.500	0.415	0.011 [0.100]	0.146 [0.094]
xanthurenic acid	0.019	4.48 × 10^−3^	0.408	0.378	0.272 [0.258]	0.437 [0.209]
L-cystathionine	0.028	1.89 × 10^−2^	0.378	0.378	0.228 [0.484]	1.000 [0.866]
homocitrulline	0.029	1.70 × 10^−2^	0.438	0.396	0.115 [0.138]	0.250 [0.183]
kynurenic acid	0.057	6.15 × 10^−2^	0.313	0.207	0.307 [0.136]	0.347 [0.100]
L-carnosine	0.061	3.52 × 10^−2^	0.441	0.438	0.183 [0.422]	0.818 [0.499]
DL-5-hydroxylysine hydrochloride	0.061	6.15 × 10^−2^	0.346	0.318	0.045 [0.059]	0.104 [0.195]
L-aminoadipic acid	0.092	9.38 × 10^−2^	0.282	0.250	0.120 [0.186]	0.240 [0.117]

## Data Availability

The data presented in this study are available upon request from the corresponding author.

## Abbreviations

The following abbreviations are used in this manuscript:

BPD	Bronchopulmonary dysplasia
EOS	Early onset sepsis
EUGR	Extrauterine growth restriction
GA	Gestational age
IUGR	Intrauterine growth restriction
LOS	Late onset sepsis
MPO	myeloperoxidase
NAD	nicotinamide adenine dinucleotide
NMDA	N-methyl-D-aspartate
NEC	Necrotizing enterocolitis
NICU	Neonatal Intensive Care Unit
PMA	Postmenstrual Age
PN	Parenteral nutrition
ROP	Retinopathy of prematurity
SGA	Small for gestational age
VLBWI	Very Low Birth Weight Infants
PCA	Principal Component Analysis
PLS2C	PLS for classification
MCC	Matthews correlation coefficient
MCCcv	Matthews correlation coefficient calculated by cross-validation
